# Sustaining control: lessons from the Lubombo spatial development initiative in southern Africa

**DOI:** 10.1186/s12936-016-1453-9

**Published:** 2016-08-12

**Authors:** Rajendra Maharaj, Devanand Moonasar, Candrinho Baltazar, Simon Kunene, Natashia Morris

**Affiliations:** 1 Office of Malaria Research, Medical Research Council, Durban, South Africa; 2 School of Health Systems and Public Health, University of Pretoria, Pretoria, South Africa; 3 School of Life Sciences, University of KwaZulu-Natal, Pietermaritzburg, South Africa; 4 Malaria Directorate, Department of Health, Pretoria, South Africa; 5 National Malaria Programme, Ministry of Health, Manzini, Swaziland; 6 Malaria Programme, Ministry of Health, Maputo, Mozambique; 7 Biostatistics and GIS, Medical Research Council, Durban, South Africa

**Keywords:** Cross-border malaria, Sustainability, Elimination

## Abstract

**Background:**

The Lubombo Spatial Development Initiative (LSDI) was a tri-country project between South Africa, Swaziland and Mozambique with the aim of accelerating socio-economic development in the region. The malaria component of the project was introduced to decrease the transmission of malaria in the region. This goal was met but with termination of this project resulted in an upsurge of malaria cases in the sub-region mainly as a result of migration from high transmission areas to low transmission ones. The movement of people across borders in southern Africa remains a challenge in sustaining malaria control and elimination.

**Methods:**

Malaria case data for Swaziland and South Africa were obtained from their respective national Malaria Information Systems. Data for Mozambique was obtained from the Mozambican Ministry of Health. Data obtained during the course of the LSDI project was compared to the case data post the termination of the LSDI.

**Results:**

The 12-year period of the LSDI showed a substantial decrease in disease burden amongst the three countries involved when compared to the baseline year of 2000. The decrease in malaria cases was 99 % in South Africa and 98 % in Swaziland. Malaria prevalence in Mozambique decreased by 85 % over the same period. However, after the LSDI ended, between 2012 and 2014, there was an upward trend in case data that was counter to the goal of elimination.

**Conclusion:**

South Africa and Swaziland benefitted from the LSDI and were able to sustain malaria control and progress to the stage of elimination. Mozambique could not sustain the gains made during the LSDI and case numbers increased. Technical and financial resources are key challenges for malaria control and elimination interventions.

## Background

**Fig. 1:**
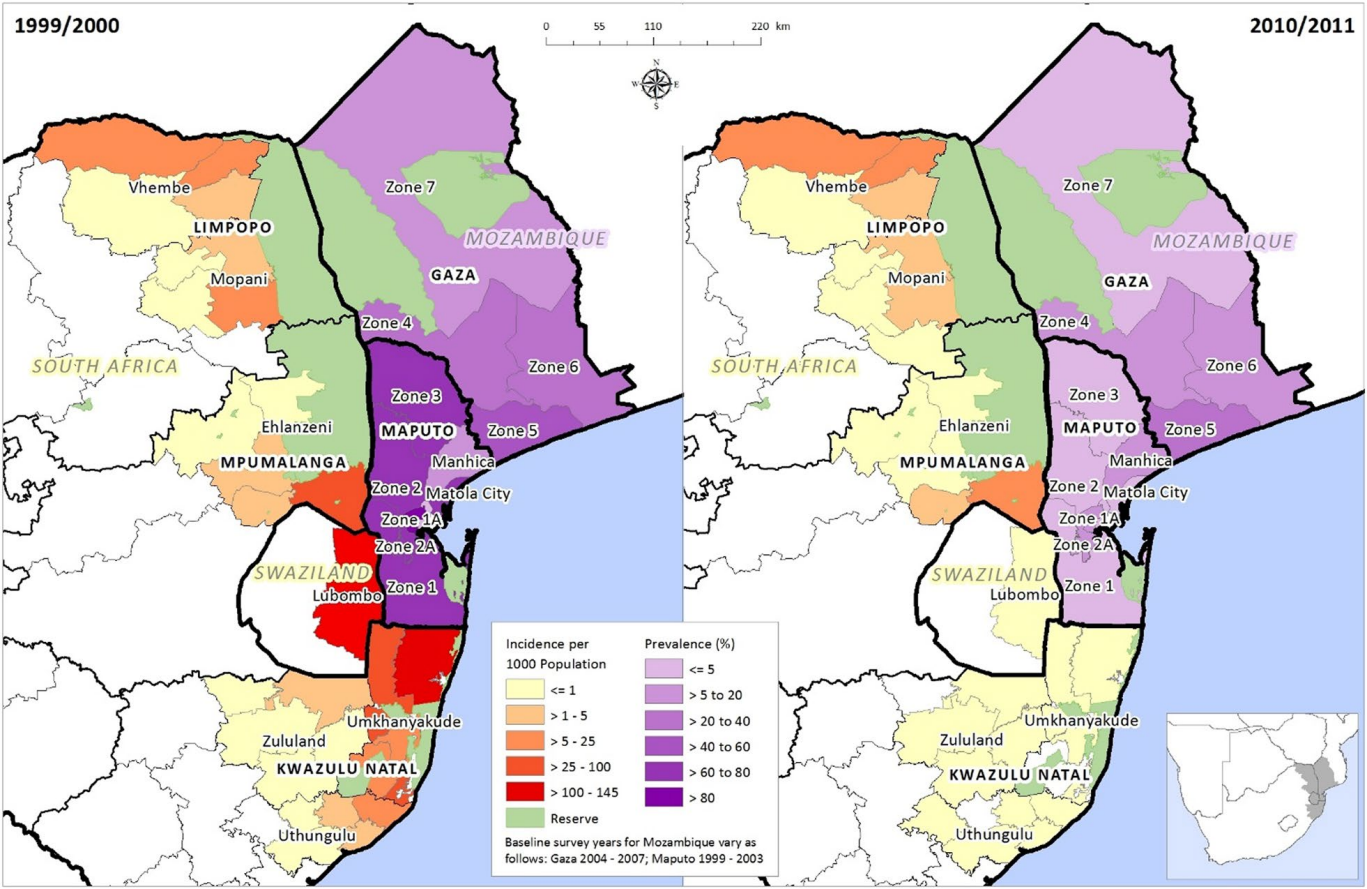
Map of the LSDI areas showing the impact of the interventions at baseline and termination

As many countries around the world move towards the goal of malaria elimination, it is becoming more evident that malaria needs to be tackled at a regional level since country efforts have not produced the desired outcome [1]. The southern African region is accelerating towards malaria elimination by 2025 and a number of regional initiatives have been established to facilitate this paradigm shift. The Elimination Eight (E8) [1] initiative aims to eliminate malaria from eight Southern African countries, including South Africa, Swaziland and Mozambique. The E8 initiative builds on the premise that harmonizing control and case management strategies across countries can help achieve elimination quicker than countries working on their own. The most successful demonstration of efffective cross-border malaria control was the Lubombo Spatial Development Initiative (LSDI), a trilateral venture between South Africa, Swaziland and Mozambique to accelerate socio-economic development in an impoverished region. This collaboration was endorsed by the Heads of State of the three countries and committed the three national malaria control programmes to work together, sharing resources and technical expertise. The area targeted for accelerated development was the northern KwaZulu-Natal Province of South Africa, eastern Swaziland and southern Mozambique (Fig. 1); areas with high malaria transmission in the three countries. A malaria control arm introduced to the socio-economic development initiative resulted in a rapid decrease in the transmission of malaria in this region [2].

The opportunity to sustain the success of the initiative was compromised as the result of financial constraints at the country level. Although the LSDI was a highly successful initiative that succeeded in reducing the malaria burden in high transmission areas of the participating countries, the financial resources that had been committed to the programme by country partners did not materialize. As a result of the insufficient funds and problems with resource mobilization, the LSDI was terminated in 2011 after 12 years of remarkable success through the implementation of vector control based solely on an indoor residual spray programme.

The implementation of the LSDI resulted in a 70 % decrease in malaria prevalence in Maputo Province, Mozambique between 1999 and 2005. The initiative also resulted in a 99 % and a 98 % decrease in the notified malaria cases in South Africa and Swaziland, respectively. Based on the impact that the LSDI had on malaria incidence in South Africa and Swaziland, the malaria control programmes in these two countries have subsequently undergone a re-orientation towards elimination. Swaziland commenced malaria elimination activities in 2011 and South Africa in 2012, around the same time that the LSDI programme ended. Similarly, Mozambique embarked on a pre-elimination agenda in Maputo Province.

This study aims to investigate the impact that the termination of the LSDI had on the countries participating in this initiative. This manuscript examines the influence of the LSDI on the malaria control programmes in the three participating countries and compare the malaria situation in these during the lifespan of the LSDI and in the 3 years immediately following its cessation.

## Methods

### Study area

The LSDI was put in place to accelerate socio-economic development of the Lubombo area through increased tourism and agricultural output [3]. Due to the high burden of malaria in the area designated for accelerated socio-economic, malaria control interventions were reinforced in the LSDI area. The area consisted of northern KwaZulu-Natal province in South Africa, the eastern Lubombo District of Swaziland, and Maputo Province in southern Mozambique. Collectively, this area is bounded by the Lubombo Mountains which lent their name to the development initiative.

### Intervention implementation

*Anopheles* vector control through indoor residual spraying (IRS) with insecticide together with parasite control through first-line treatment with artemisinin-based combination therapy (ACT) were the two key malaria control interventions implemented.

In Mozambique, twice annual IRS with bendiocarb insecticide (Bayer CropScience, Mannheim, Germany) was introduced in Maputo Province in 2000. In South Africa, IRS with DDT was reintroduced in KwaZulu-Natal Province in 2000 after the detection of pyrethroid resistance. Pyrethroid insecticide continued to be used in homes with painted walls due to the visible residues of DDT on such surfaces. In Swaziland, DDT was used in traditional structures and pyrethroids were used for western type structures. In all areas the spray coverage was greater than 80 %.

Prior to the introduction of artemisinin-based combination therapy, chloroquine and sulfadoxine–pyrimethamine (SP) had been the first- and second-line treatments, respectively, in both Swaziland and Mozambique, whereas in South Africa, sulfadoxine–pyrimethamine had been the first-line treatment. In KwaZulu-Natal, artemether–lumefantrine was introduced in February 2001 and in the LSDI areas of Swaziland it was introduced in 2010. The phased implementation of artesunate plus SP commenced in Maputo Province in 2004.

### Data collection

Malaria cases confirmed at health facilities across the three South African malaria-endemic provinces were entered into a clinic or hospital case register. Individual case records are also routinely entered onto malaria notification forms, which were submitted on a weekly basis to provincial malaria control programmes (MCP). At the MCP offices, individual case data including patient details, symptoms, diagnosis, microscopy and/or rapid diagnostic test (RDT) results, treatment administered, referrals information, the locality the patient resided in and the reporting health facility’s name were entered into a computerized malaria information system. South Africa and Swaziland started classifying malaria as local or imported in 2011 and 2010 respectively, at the national level.

### LSDI data

The number of malaria cases for Swaziland and South Africa were obtained from their national Malaria Information Systems (MIS) since malaria is a notifiable disease in the participant countries. The MIS was designed to document all malaria cases notified by health facilities, and in the case of South Africa, includes cases actively detected during response by field staff to malaria outbreaks or while conducting random household visits and in follow-up of confirmed cases.

Prevalence data was utilized from southern Mozambique from 1999 to 2011. At each of 26 sentinel sites, cross-sectional parasite surveys were performed on a random sample of 120 individuals ≥2–15 years of age. Sentinel sites were each divided into localities from which participants were selected to ensure as much geographical spread as feasible. Rapid diagnostic tests were used to assess prevalence of *Plasmodium falciparum* infection. Prevalence was calculated annually for each district.

### Post-LSDI data

#### South Africa

Malaria data was once again obtained from the MIS, a system that records all the malaria cases in the country.

#### Swaziland

Data was provided by the Ministry of Health. Missing malaria case data was obtained from the World Malaria Reports of 2013, 2014 and 2015 [4–6]. Population data was obtained from the World Population Prospects 2015 [7] in order to calculate malaria incidence rates.

#### Mozambique

The 2012–2014 malaria incidence data for Mozambique was obtained from the National Malaria Control Programme of the Mozambican Ministry of Health.

### Data analysis

Descriptive statistics were used to analyse the trend in malaria transmission post-LSDI. The proxy for the success of the LSDI was the decrease in the incidence of malaria in Mozambique, South Africa and Swaziland. Therefore, the indicators for the current situation will consist of incidence in South Africa and Swaziland (2011–2013), and incidence data for southern Mozambique (2012–2014).

## Results

### Achievement of the LSDI

Compared with the baseline year of 2000, the success of the LSDI is evidenced in the substantial decreases in disease burden observed over a 12-year period across the three participating countries (Fig. 1). In South Africa, the incidence of the disease decreased by 99 % compared with the epidemic year of 2000, whilst in Swaziland the burden of the disease decreased by 98 % when compared to the 2000 figures.

**Fig. 2:**
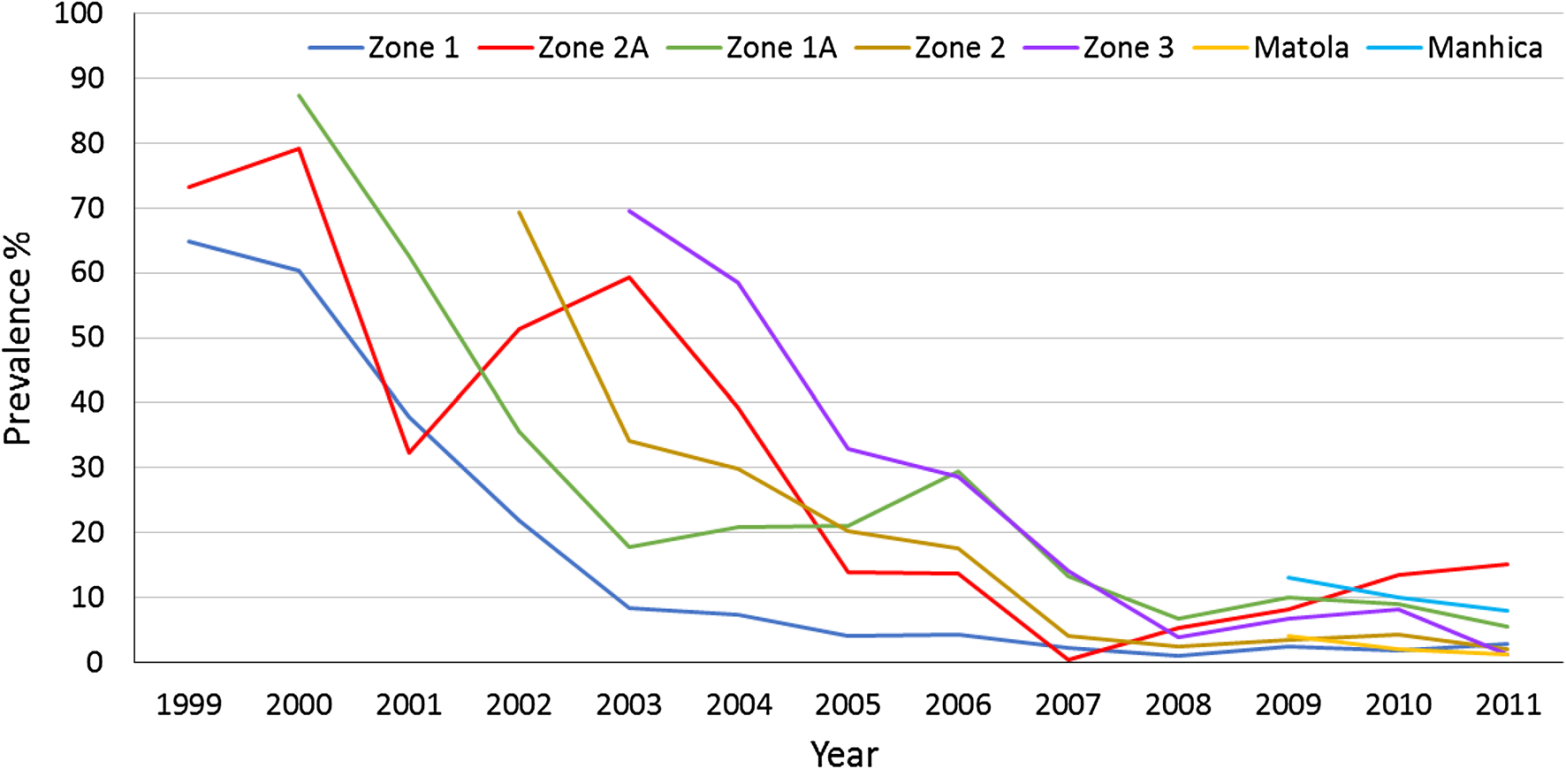
The decrease in prevalence in each zone of Maputo Province

Due to the lack of accurate incidence data in Mozambique, reliance was made on prevalence data for children 2 to <15 years old, to determine impact of the malaria control interventions. Since the interventions were introduced gradually over a number of years, baseline surveys were carried out in each of the implementation zones immediately before indoor residual spraying could be instituted. Malaria prevalence decreased in all zones from an average of 70 % to an average of 5 % during the lifespan of the LSDI (Fig. 2).

### Impact of the termination of the LSDI

#### Mozambique

Unfortunately, the gains achieved in Mozambique could not be sustained following termination of the LSDI programme. Since there were no coordinated vector control measures implemented after the conclusion of the LSDI, malaria case numbers increased. When comparing the increases in cases, the 2014 data was compared to the period when malaria data was available for Maputo province (2012). It is apparent that from 2012 to 2014 there was a considerable increase in the number of cases reported in all districts (Table 1). All districts showed increases in cases reported in 2014 compared to 2012. Therefore, in Maputo Province there was an overall increase of 50 % in cases between 2012 and 2014.

**Table 1:** Case data from Mozambique for the period succeeding the LSDI

Districts	Number of cases			% change
	2012	2013	2014	
Boane	16,392	23,905	24,927	52.07
Magude	9845	14,702	13,661	38.76
Manhica	49,206	52,733	65,578	33.27
Marracuene	7471	13,375	26,045	248.61
Matutuine	855	927	1446	69.12
Moamba	9967	8472	16,856	69.12
Namaacha	1960	4937	8175	317.09
Matola city	32,377	35,819	36,870	13.88
Total	128,073	160,270	192,558	50.35

#### Swaziland

**Fig. 3:**
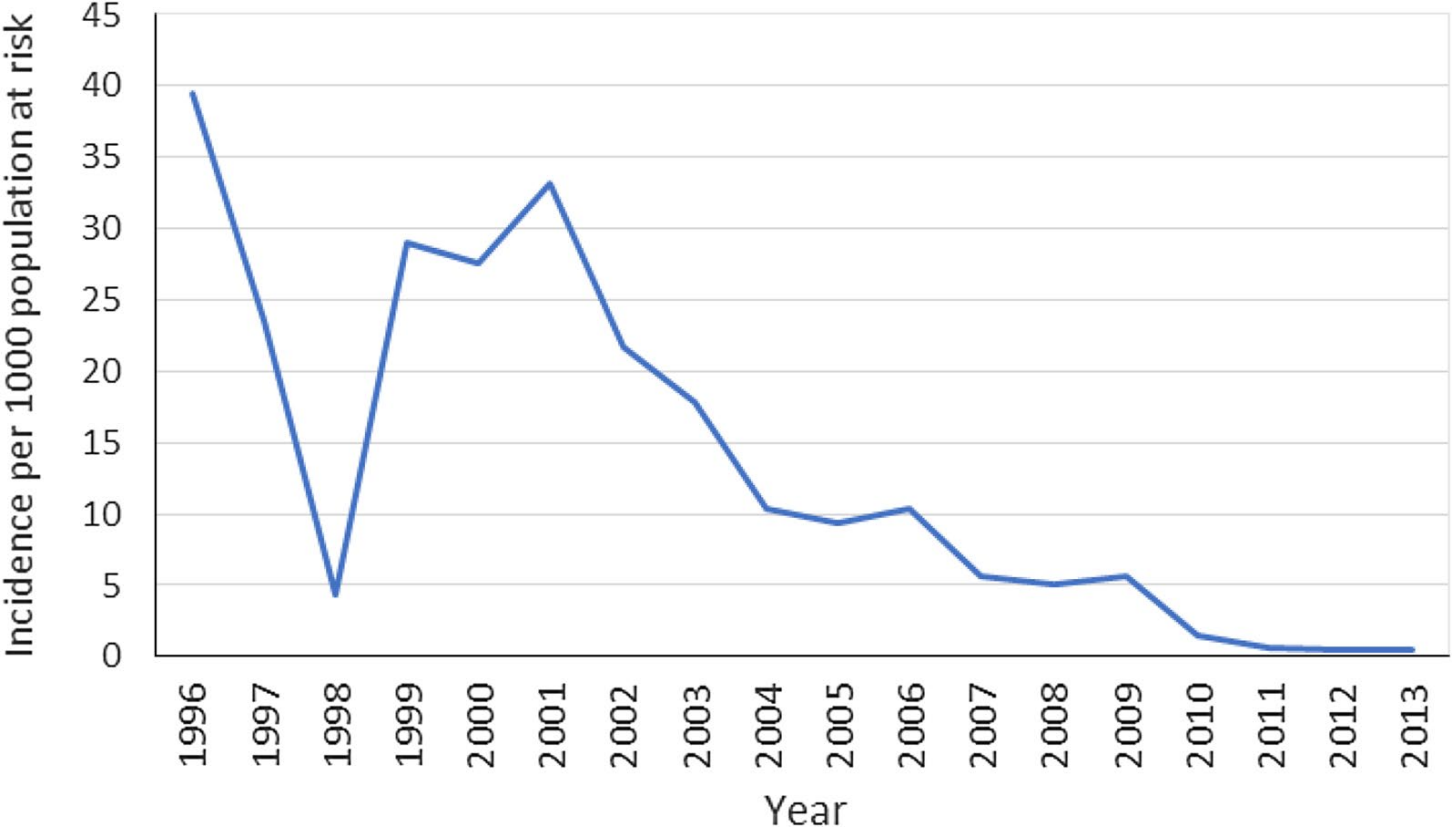
Incidence data for Swaziland from 1996 to 2013

This country benefitted from the LSDI and recorded significant decreases in malaria incidence during the lifespan of the initiative. Although Swaziland retained its malaria control programme when the LSDI ended, the number of cases increased post-LSDI but was curtailed by increased interventions in 2014 (Fig. 3). From 2012 to 2013 the number of actual cases reported increased from 626 in 2012 to 962 in 2013. Since Swaziland has adopted an elimination agenda and was able to respond to the increases in cases; only 188 of the 711 cases (26.4 %) reported in 2014 were locally acquired.

Swaziland’s goal was to eliminate malaria by the end of 2015, but even this country with very low transmission of malaria experienced an upsurge of malaria. The incidence of the disease reduced from 40 per 1000 population to 0.5 cases per 1000 by 2011 (Fig. 3). In 2013, following closure of the LSDI, malaria incidence increased marginally to 0.8 per 1000 population. This increase in the incidence of the disease is certain to hamper Swaziland’s goal to eliminate malaria; already the target date of eliminating malaria by 2015 has passed. One of the major problems experienced by the malaria control programme in Swaziland is the importation of malaria from neighbouring countries, particularly Mozambique.

**Fig. 4:**
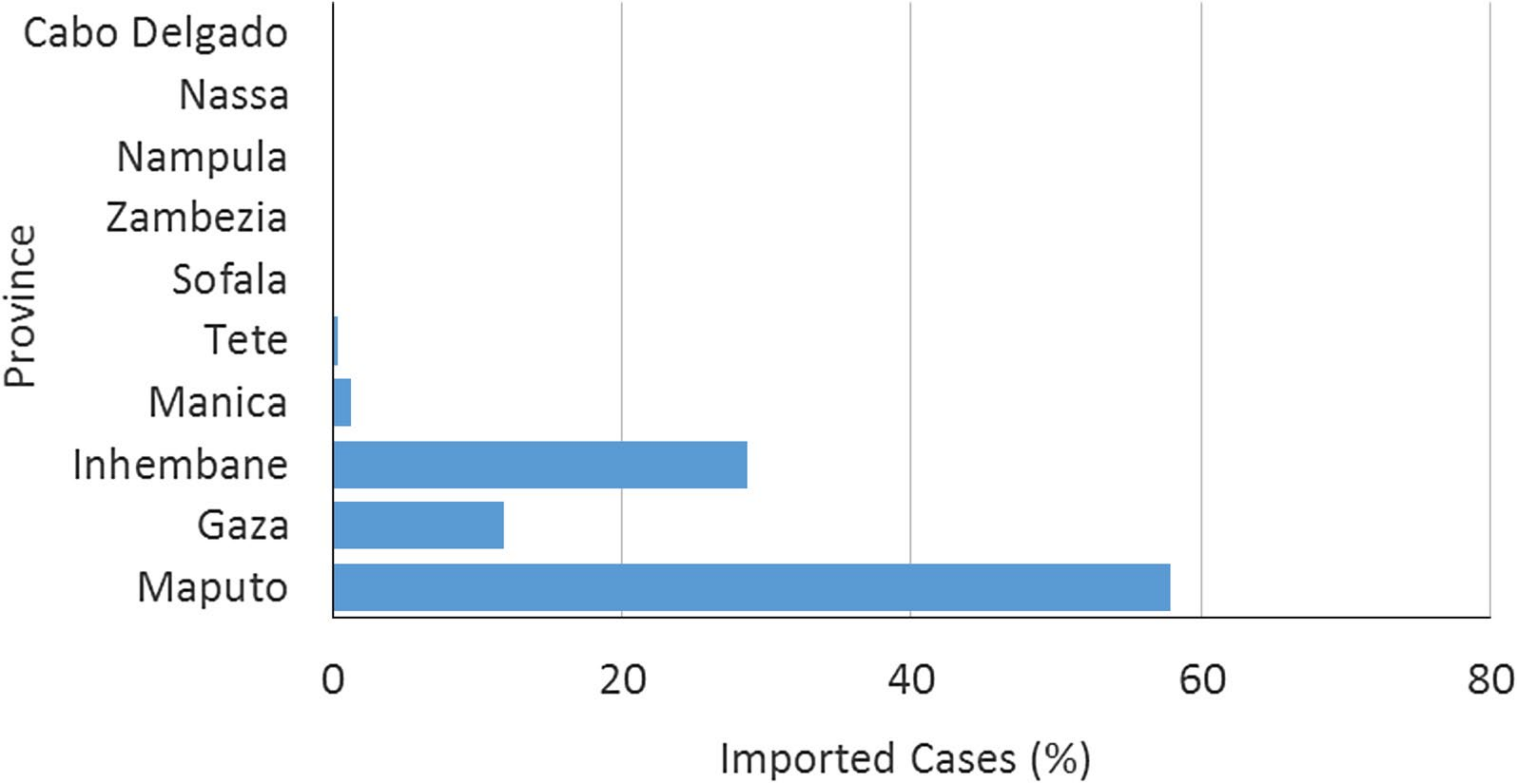
Mozambiquan origin of imported cases into Swaziland

The largest proportion of imported malaria into Swaziland originates from southern Mozambique, most noticeably from the three southern-most provinces of Maputo, Gaza and Inhambane. The bulk of importation originates from Maputo Province which shares a border with Swaziland (Fig. 4). This area previously benefitted from the malaria control interventions implemented as part of the LSDI.

#### South Africa

**Fig. 5:**
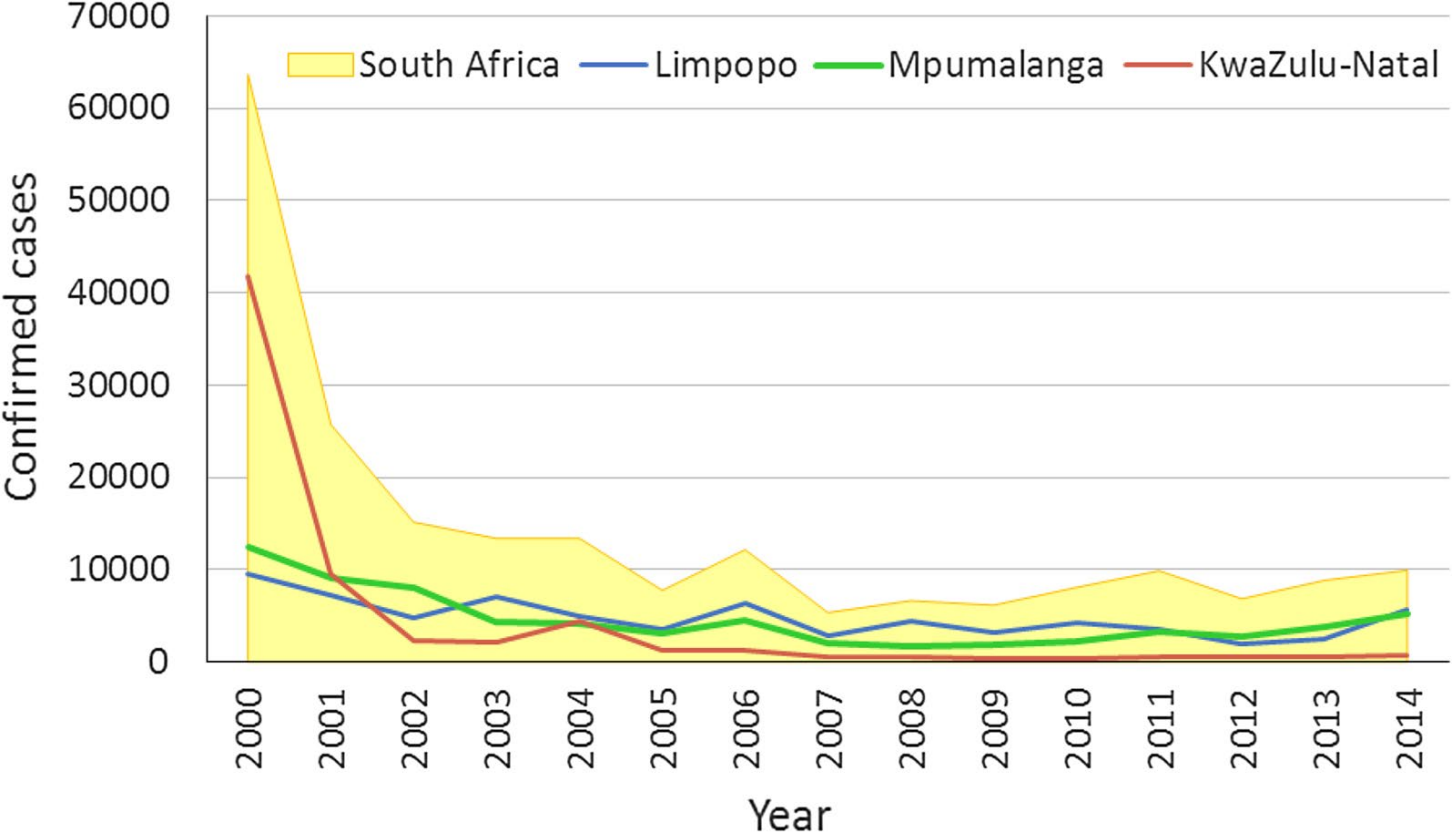
Trend of malaria cases at a national and provincial level

South Africa benefitted immensely from the LSDI interventions in place in the participating countries. Since the start of the LSDI, which coincided with the 1999/2000 epidemic, there were sizeable decreases in the incidence of malaria in the country. From 2007 to 2011, the total number of cases in the country remained below 10,000 (Fig. 5). However, in 2012 the number of cases in the country as a whole began to increase and, in 2014, increased to over 10,000 for the first time in 8 years. Both Limpopo and Mpumalanga Provinces reported increases in case numbers between 2012 and 2014. Case numbers in KwaZulu-Natal have remained consistently low since 2007.

**Fig. 6:**
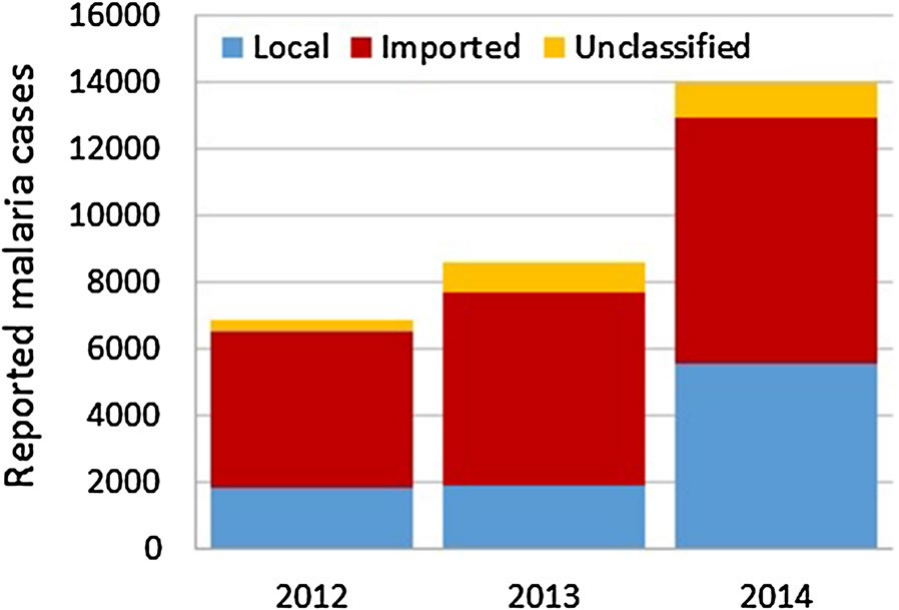
The different categories of malaria case data for South Africa

An interrogation of the imported malaria data for South Africa showed that the number of imported cases in the country increased between 2012 and 2014 (Fig. 6). The number of imported malaria cases increased with the lengthening of time since the last LSDI spray activities concluded in 2011.

## Discussion

The development of a regional malaria control programme under the umbrella of the LSDI saw a harmonized policy for vector control and treatment of infected patients being implemented in the participating areas. Over a period of 10 years, this initiative resulted in the decrease of malaria incidence in South Africa and Swaziland by 99 % overall, and a decrease in the prevalence of the disease in Maputo Province to less than 5 %.

However, in 2011 (12 years after commencement of the LSDI) the programme was terminated. The LSDI was mainly funded by international donors and when funding ceased, the interventions in southern Mozambique could not be continued and the co-ordinated implementation of vector control activities came to an end. At this time, the Ministry of Health in Mozambique was not in a position to maintain the malaria control interventions that were put in place during the tenure of the LSDI, as had initially been intended. As a result there was no co-ordinated or sustained implementation of the vector control interventions that had achieved remarkable gains over the previous decade. Whilst Mozambique was not able to sustain the indoor residual spray programme that was implemented as part of the LSDI vector control programme, South Africa and Swaziland reverted to country specific programmes. Furthermore, these two countries had very successfully decreased their disease burden during the preceding years such that they were able to embark on an elimination agenda.

Although, South Africa and Swaziland embarked on elimination programmes, the end of the LSDI impacted on the goal to achieve elimination. In both these countries, the malaria cases and deaths began to increase after the LSDI had ended and this was linked mainly to the loss of control in Mozambique as the control activities in South Africa and Swaziland were well maintained post-LSDI with South Africa and Swaziland achieving a greater than 80 % spray coverage in areas targeted by IRS. The success of the Swaziland malaria programme over the last decade has raised expectations for malaria elimination. However, with the movement of people between Swaziland and Maputo Province in Mozambique, imported cases proved to be a challenge for the elimination programme of Swaziland. Swaziland had hoped to achieve elimination by 2015, but the goal was not met. Similarly, in South Africa the target date for malaria elimination is 2018 but the increase in cases and deaths observed since 2012 will prove challenging to the country’s elimination goal.

The implementation of cross border malaria control is highly achievable as has been demonstrated by the LSDI. The implementation of cross border collaboration through the LSDI has been very successful and stands as an example of best practice on the African Continent. The LSDI very effectively demonstrated the impact of coordinated vector control and case management in a high transmission setting. Over a 12-year period, the prevalence and incidence of the disease was dramatically reduced but a year after the interventions were discontinued, the number of cases began to increase. This was counter to the elimination agenda and prevented Swaziland from meeting its target. Although a sustainability plan was developed as part of the LSDI roll-out, the Ministry of Health in Mozambique was unable to maintain the gains of the LSDI when the initiative came to a premature end. This was mainly due to financial constraints at a country level. Therefore, the loss of sustainability in implementing malaria control interventions impacted negatively on the elimination agendas of South Africa and Swaziland. Mozambique saw a rapid increase in the number of cases reported from Maputo Province due the withdrawal of the highly effective vector and parasite control interventions that were implemented as part of the LSDI programme. In South Africa and Swaziland, the elimination efforts were disrupted primarily by the movement of infected individuals into these countries. There was an upsurge in the number of imported cases being reported in these countries that influenced their status in the elimination continuum.

Failure to sustain the malaria control interventions encouraged resurgence of the disease in areas that had been well controlled. Most of the resurgence in South Africa and Swaziland was due to human movement and imported malaria, Sturrock et al. [8] state that the failure of the Global Malaria Eradication Programme (GMEP) to eliminate malaria in the 1950s was due to the importation of malaria infections. As we have seen in Swaziland and as highlighted by Sturrock et al. [8] imported infections are of increasing importance in countries targeting elimination. Imported cases can reintroduce parasites into areas previously free of transmission; maintain hotspots of transmission; introduce drug resistant strains, and pose challenges to national malaria control programmes [9].

Importation of infection drives local transmission of malaria. Russel et al. [10] reiterated the lessons learned from the GMEP in that with its collapse, malaria cases in Africa and Asia steadily increased and even surpassed pre-intervention levels. IRS programmes became ineffective in many parts of the world when mosquito vector populations quickly rebounded to pre-spray levels within a few years despite continual spraying pressure.

Malaria control strategies, policies and timing of interventions may differ across national borders, making cross-border collaboration challenging. Harmonized policies, timing of spray activities, sharing of resources are required for cross-border collaboration [11]. Nonetheless, the vision of cross-border malaria control establishes an avenue for developing harmonized drug and insecticide policy between neighbouring countries that align with national efforts [12]. The development and synchronous implementation of interventions across borders assists in the implementation of vector control and case management measures that decreases insecticide and drug pressure, thereby slowing the onset of resistance. Once harmonized policies are disregarded, insecticide and drug pressure on both vectors and parasites will increase thereby fuelling programme failure.

Thus the premise of Pindolia et al. [9] that it is essential for control planning to identify human and parasite movement since imported infections can reintroduce infections into areas previously free of disease is relevant to the regional initiative. In endemic areas within South Africa, hotspots have developed and may be sustained through imported malaria as well as local transmission. Swaziland is reporting increased imported malaria that may be driving its local transmission. With the termination of the LSDI, the harmonized policies that had been developed and implemented in the region were no longer enforced, particularly in Mozambique. Vector control is essential to reducing malaria transmission and has not been effectively implemented in Mozambique in recent years, due to a range of technical and administrative factors [13]. As a result, there was a huge increase in cases in Maputo Province where the prevalence had previously been reduced drastically. The resurgence of malaria in Mozambique was as attributed in part to the cessation of malaria control and was attributed to the financial resource constraint [14]. From Maputo province, human population movement of infected individuals carried the disease to neighbouring countries. The demise of the implementation of harmonized policies derailed the elimination agenda in South Africa and Swaziland as case numbers began to increase. The termination of the LSDI has had a negative impact on the malaria burden of the three countries.

## Conclusion

While cross-border collaboration should be considered early in the control stage, its importance in managing re-importation is highly evident in the elimination stage. This is particularly critical in areas with significant population movement from areas of high transmission intensity. A good understanding of malaria epidemiology in the various transmission foci and of their physical characteristics will be critical for targeting the elimination programme activities in South Africa and Swaziland as well as bordering areas of Mozambique, and for advancing towards zero transmission in a safe, cost-effective and an efficient manner.

On a regional scale, the LSDI programme demonstrated that malaria elimination is only possible if all countries in a region embark on activities that strengthen their elimination campaign. As elimination progresses in South Africa and Swaziland, it paves the way for elimination in Mozambique and Zimbabwe. However, it should be borne in mind that the reverse is also true. As has been demonstrated by the implementation of the LSDI malaria control initiaitve in countries with contiguous borders, gains made over years can be lost in a matter of months if there is no sustainability of interventions in any of the partner countries. Before embarking on a large-scale project such as the LSDI it is critical to ensure that there is adequate funding to sustain the gains made.

## Abbreviations

ACT: artemisinin-based combination therapy
GIS: geographical information system
GMEP: Global Malaria Eradication Programme
IRS: indoor residual spraying
LSDI: Lubombo spatial development initiative
MCP: malaria control programme
MIS: malaria information system
RDT: rapid diagnostic test
SP: sulfadoxine–pyrimethamine
